# Theoretical Insights on ORR Activity of Sn-N-C Single-Atom Catalysts

**DOI:** 10.3390/molecules28145571

**Published:** 2023-07-21

**Authors:** Yuhui Zhang, Boyang Li, Yaqiong Su

**Affiliations:** 1 School of Energy and Chemical Engineering, Xiamen University Malaysia, Sepang 43900, Malaysia; ege2009279@xmu.edu.my; 2 School of Chemistry, Engineering Research Center of Energy Storage Materials and Devices of Ministry of Education, National Innovation Platform (Center) for Industry-Education Integration of Energy Storage Technology, Xi’an Jiaotong University, Xi’an 710049, China; liby22@stu.xjtu.edu.cn

**Keywords:** N-doped carbon, single Sn atom, oxygen reduction reaction, electrocatalysis

## Abstract

The advancement of efficient and stable single-atom catalysts (SACs) has become a pivotal pursuit in the field of proton exchange membrane fuel cells (PEMFCs) and metal-air batteries (MABs), aiming to enhance the utilization of clean and sustainable energy sources. The development of such SACs has been greatly significant in facilitating the oxygen reduction reaction (ORR) process, thereby contributing to the progress of these energy conversion technologies. However, while transition metal-based SACs have been extensively studied, there has been comparatively less exploration of SACs based on p-block main-group metals. In this study, we conducted an investigation into the potential of p-block main-group Sn-based SACs as a cost-effective and efficient alternative to platinum-based catalysts for the ORR. Our approach involved employing density functional theory (DFT) calculations to systematically examine the catalyst properties of Sn-based N-doped graphene SACs, the ORR mechanism, and their electrocatalytic performance. Notably, we employed an H atom-decorated N-based graphene matrix as a support to anchor single Sn atoms, creating a contrast catalyst to elucidate the differences in activity and properties compared to pristine Sn-based N-doped graphene SACs. Through our theoretical analysis, we gained a comprehensive understanding of the active structure of Sn-based N-doped graphene electrocatalysts, which provided a rational explanation for the observed high four-electron reactivity in the ORR process. Additionally, we analyzed the relationship between the estimated overpotential and the electronic structure properties, revealing that the single Sn atom was in a +2 oxidation state based on electronic analysis. Overall, this work represented a significant step towards the development of efficient and cost-effective SACs for ORR which could alleviate environmental crises, advance clean and sustainable energy sources, and contribute to a more sustainable future.

## 1. Introduction

The growing attentions toward energy demand and environmental issues have made proton exchange membrane fuel cells (PEMFCs) and metal-air batteries (MABs) promising candidates, given their highly efficient and clean energy conversion and storage devices which can convert hydrogen into electrical energy in a sustainable and environmentally friendly way [1,2]. However, the oxygen reduction reaction (ORR) occurring at the cathode in PEMFCs has been observed to be comparatively slow and therefore diminish the efficiency of energy transformation, which poses a major challenge for researchers. Platinum-group metal-based catalysts have been recognized for their excellent catalytic performance in the ORR. However, their limited availability and high cost impose constraints on their broader utilization [3]. Alternatively, main-group p-block metals such as Sn, which possess closed d-shells, have exhibited promising potential as active sites for the ORR. These metals offer advantages such as lower cost and abundant reserves, making them attractive alternatives to platinum-group metals [4]. In recent years, there has been a surge of interest in single-atom catalysts (SACs) within the field of heterogeneous catalysis. These catalysts have garnered significant attention due to their exceptional properties, including maximum atomic utilization, unique electronic structures, and unsaturated coordination environments. SACs have emerged as a promising avenue for addressing the limitations of traditional catalysts, such as poor atom efficiency and undesired side reactions [2]. One of the key advantages of SACs is their ability to maximize the use of catalytically active metal atoms. By dispersing metal atoms as single entities on a support material, SACs avoided the formation of metal nanoparticles or clusters, which often suffered from low atomic utilization and susceptibility to sintering or leaching. This atomic-level dispersion not only enhanced the efficiency of catalytic processes but also enabled precise control over catalytic sites and reactivity. The electronic structure of SACs is another significant aspect that contributes to their remarkable catalytic performance. The isolation of metal atoms in SACs leads to unique electronic properties, such as enhanced electron transfer and modified electronic states. These features can influence the adsorption, activation, and transformation of reactant molecules, ultimately influencing the overall catalytic activity and selectivity. Furthermore, the unsaturated coordination environment of SACs, typically achieved by supporting metal atoms on defect-rich or functionalized materials, offers increased accessibility of active sites and promotes desirable catalytic reactions. The introduction of nitrogen doping, for instance, can modulate the surface properties of carbon materials, leading to enhanced metal–support interactions and improved catalytic performance [5]. Given their numerous advantages, SACs have been extensively explored in various catalytic applications, including electrocatalysis, photocatalysis, and heterogeneous catalysis. They have demonstrated remarkable activity and selectivity in diverse reactions. Notably, Sn-based SACs have demonstrated remarkable catalytic activity in various chemical reactions [6]. For instance, Liang et al. conducted a systematic study on the effects of altering the nitrogen coordination number in Sn single-atom catalysts (SACs) to investigate the activity and selectivity of CO_2_ hydrogenation to formic acid (HCOOH) [7]. Tang et al. conducted a comprehensive theoretical investigation on the performance of Sn single-atom catalysts (SACs) in the oxygen reduction reaction (ORR) using an associative four-electron mechanism [8]. However, the inherent high surface energy of Sn atoms made them prone to agglomeration, resulting in a significant decline in their catalytic activity for the ORR [9]. To address this challenge, precise control over the interaction between the single-metal atom and the support was crucial. Therefore, the use of an appropriate support that can effectively stabilize metal atoms and create highly stable active sites is of utmost importance in the development of SACs [10]. Additionally, the physicochemical properties of the support material significantly influenced the overall catalytic performance of SACs. In this regard, nitrogen-doped graphene has garnered considerable attention as an excellent support material for the synthesis of SACs. Nitrogen doping introduces nitrogen atoms into the graphene lattice, leading to several advantageous features. Firstly, nitrogen-doped graphene exhibited a high specific surface area, providing ample surface sites for anchoring and dispersing individual metal atoms [11]. This maximizes the exposure of active sites and enhances catalytic efficiency. Moreover, nitrogen-doped graphene possesses a well-defined pore distribution, which further enhances its catalytic performance. The presence of pores facilitates the diffusion of reactant molecules and promotes their interaction with the anchored metal atoms. This not only improves the accessibility of active sites but also enhances the adsorption and activation of reactants, leading to enhanced catalytic activity. Furthermore, the incorporation of nitrogen into the graphene structure results in modifications to its electronic properties. Nitrogen atoms can act as electron donors or acceptors, leading to changes in the local electronic environment around the anchored metal atoms [12]. This modification of the electronic structure can influence the adsorption energies of reactants, facilitate charge transfer processes, and modulate the reaction kinetics, ultimately enhancing the overall catalytic performance of SACs. These characteristics provided abundant sites for anchoring single atoms and facilitate the efficient transformation of reactants [13]. In addition, the nitrogen atoms presented in this support can effectively coordinate with single-metal atoms, forming M-Nx moieties that are regarded as the primary catalytic centers in nitrogen-doped graphene-based SACs [14,15]. This distinctive coordination environment not only enhances the activity of the catalysts by virtue of their specific electronic structure but also serves to securely anchor the metal atoms, preventing their aggregation and leaching during both the preparation and reaction processes. Moreover, the utilization of N-doped graphene as a modified support for anchoring single transition metal atoms has been extensively investigated and proven to be effective in facilitating the ORR process. However, there were still unresolved issues regarding the anchoring of main-group metal Sn on N-modified carbon. Therefore, the combination of the p-block metal Sn as the primary active site and N-doped graphene as the supporting material (Sn-N-C) holds significant potential for various catalyst research applications. For instance, Strasser et al. developed an electrocatalyst by anchoring the single Sn atom on the N-doped carbon matrix, which exhibited excellent catalytic performance in the four-electron pathway of ORR [16]. The highly dispersed Sn-N-C SACs also exhibited superior activity in CO_2_ reduction reaction and lithium–sulfur batteries [17]. Outstanding catalytic performance, cost-effectiveness, and the non-toxic nature of N-doped graphene Sn-SACs are expected to significantly enhance the ORR performance in PEMFCs, thereby promoting sustainable advancements in this field. Although the catalytic properties and ORR mechanism of Sn-based single-atom catalysts (Sn-SACs) have been extensively explored using advanced experimental characterizations, certain unresolved issues persist. These issues, which may involve aspects such as electronic structure, reaction energy profiles, and the impact of heteroatom decoration on Sn-SACs and their electrocatalytic activity, can be effectively addressed using theoretical calculation approaches.

In this study, we employed density functional theory (DFT) calculations to systematically investigate the electronic structure, reaction mechanisms, and catalytic activities of the ORR over Sn-based N-doped graphene SACs. Furthermore, to better illustrate the difference in the ORR performance of Sn-based SACs, we conducted a comparative analysis between catalysts with H-decorated N atoms and those without H-decoration. Firstly, we constructed structural models of Sn-based SACs by anchoring an Sn single atom onto pristine N-doped graphene and H-decorated N-doped graphene, forming the SnN_4_ and SnN_4_-H configurations, respectively. Formation energy analysis revealed the structural stability of both SnN_4_ and SnN_4_-H. Subsequently, we investigated the adsorption of various species on SnN_4_ during the ORR and delved into the detailed ORR mechanisms. The electrocatalytic performance and electronic structural properties of these two catalysts were extensively discussed. Notably, SnN_4_ exhibited a lower overpotential for ORR compared to its H-decorated counterpart. To elucidate the correlation between the ORR activity and Sn catalysts, we conducted density of states (DOS) and Bader charge analyses. The insights obtained from this study hold the potential to serve as a reliable theoretical foundation, accelerating the development of high-performance and stable catalysts for ORR in PEMFCs, thereby advancing the utilization of clean and sustainable energy sources.

## 2. Results and Discussion

**Figure 1:**
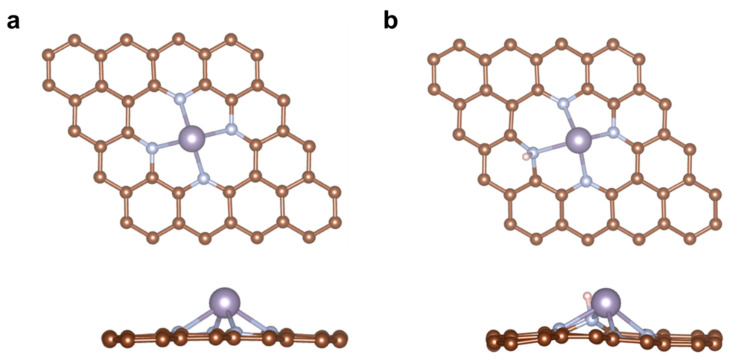
The top and side view of SnN_4_ (**a**) and SnN_4_-H (**b**). Color code: brown, C; cyan, N; purple, Sn; white, H.

**Figure 2:**
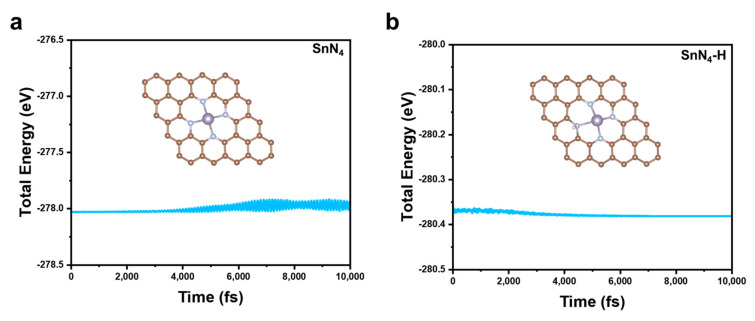
The AIMD simulation of SnN_4_ (**a**) and SnN_4_-H (**b**) with 1 fs time step under 300 k for 10 ps. The inset in the figure represents the structures after AIMD simulation.

Based on previous research, nitrogen doping in graphene can be categorized into three types: pyridinic N, graphitic N, and pyrrolic N [5]. Experimental evidence suggested that pyridinic N exhibited high reactivity towards oxidation and reduction reactions, and the N-doped carbon framework was relatively stable under reaction conditions [18]. Therefore, this work focused on pyridinic nitrogen-doped graphene as the support for the Sn-SACs, and the coordination of N atoms was set as 4 according to the experimental characterization [19]. Figure 1a depicts the top and side view of a graphene substrate hosting a pyridinic Sn-N-C catalyst, with a single Sn atom occupying the center of a carbon vacancy, N atoms replacing four adjacent carbon atoms to form pyridinic N, then coordinating with a single Sn atom to form a Sn-N_4_ structure. Due to the large atomic radius of an Sn atom, a local bulge was created in the N-doped graphene matrix, and the average length of Sn-N bonds was 2.26 Å. In contrast, H-decorated catalyst SnN_4_-H was constructed by adsorbing an H atom onto pyridinic N (Figure 1b). The average length of the Sn-N bond was 2.29 Å, which indicated a slight extension of the Sn-N bond after H decoration. Due to their high surface energy, SACs can easily aggregate into clusters or nanoparticles. Therefore, the stability of the two Sn-SACs were analyzed via their binding energy to pristine and H-decorated N-doped graphene. The calculated E_b_ of SnN_4_ and SnN_4_-H was −5.85 eV and −4.81 eV, respectively. This result showed that both catalysts had E_b_ below 0 eV, indicating that the single Sn atom can be stably anchored on the N-doped graphene matrix. Moreover, the high surface energy of Sn atoms predisposes them to agglomerate into nanoclusters or particles. To address this issue, we conducted further investigations to assess the thermodynamic stability by comparing the binding energy with the bulk cohesive energy (E_coh_) of Sn, which was calculated to be −4.09 eV. Intriguingly, the binding energy (E_b_) of both SnN_4_-H and SnN_4_ was found to be smaller than E_coh_, indicating effective prevention of Sn atom aggregation and stable anchoring on both pristine and H-decorated N-doped graphene surfaces. Additionally, we investigated the adsorption of single Sn atoms on the graphene matrix (Sn-gra), which exhibited a higher E_b_ (Table S1) compared to the E_b_ of SnN_4_ and SnN_4_-H, suggesting an unfavorable diffusion of Sn into the carbon matrix. To assess the dynamic stability of SnN_4_ and SnN_4_-H, ab initio molecular dynamics (AIMD) simulations were performed using the Nosé–Hoover thermostat model implemented in VASP. The simulations were conducted in the canonical ensemble at 300 K for a duration of 10 ps with a time step of 1 fs. As depicted in Figure 2, the system energy of both structures remained stable during the 10 ps simulation at 300 K. The stable structures after the simulation were shown in the inset. Although the energy of the SnN_4_ system exhibited minor fluctuations ranging from −278.02 eV to −277.91 eV, during the latter half of the 10 ps simulation, the overall system remained stable. Similarly, for the SnN_4_-H system, there were subtle energy fluctuations from −278.35 eV to −278.38 eV in the initial 3 ps, after which the system gradually stabilized. The RMSD (root mean square deviation) analysis was further investigated and exhibited in Figure S1, and the variation trends were consistent with the energy analysis and AIMD results obtained for the two systems. These results demonstrated that the SnN_4_ and SnN_4_-H systems exhibited high thermodynamic stability at 300 K.

Furthermore, we investigated the adsorption strength of O_2_ on the two Sn-based catalysts, as the initial adsorption of O_2_ was a crucial step preceding the electrochemical ORR. By examining the O_2_ adsorption behavior, we can gain insights into the catalyst’s ability to facilitate the subsequent ORR process. Understanding the adsorption strength of O_2_ provides valuable information for optimizing the catalyst’s performance, enhancing its catalytic activity, and improving the overall efficiency of the ORR system. The corresponding adsorption structure is exhibited in Figures S2 and S3. The obtained results indicated that both catalysts exhibited a chemisorption state of O_2_, and the bond length between Sn and O was 1.88 Å for SnN_4_ and for 1.86 Å SnN_4_-H. Compared with the O-O bond length of the gas phase (1.23 Å), the O-O bond length (1.26 Å and 1.29 Å) on the catalysts were significantly lengthened, which indicated the O_2_ had been activated. The adsorption energy of SnN_4_ and SnN_4_-H were −0.45 eV and −0.49 eV, respectively. This result demonstrated that the ability of oxygen adsorption on SnN_4_-H was slightly stronger than on SnN_4_. Furthermore, we investigated the electrocatalytic reduction product of H_2_O using the typical associative four-electron (4e^−^) pathway of ORR. The detailed elementary reaction steps involved in all four-electron associative pathways of ORR in our work are shown in the following four equations:

(1) $$*+O_{2}^{}+H^{+}+e^{-}\overset{}{↔}OOH^{*}$$

(2) $$OOH^{*}+H^{+}+e^{-}\overset{}{↔}O^{*}+H_{2}O$$

(3) $$O^{*}+H^{+}+e^{-}\overset{}{↔}OH^{*}$$

(4) $$OH^{*}+H^{+}+e^{-}\overset{}{↔}*+H_{2}O$$

where * denotes the active site of electrocatalyst, and OH*, O*, and OOH* were the adsorbed intermediates of the ORR [20].

**Figure 3:**
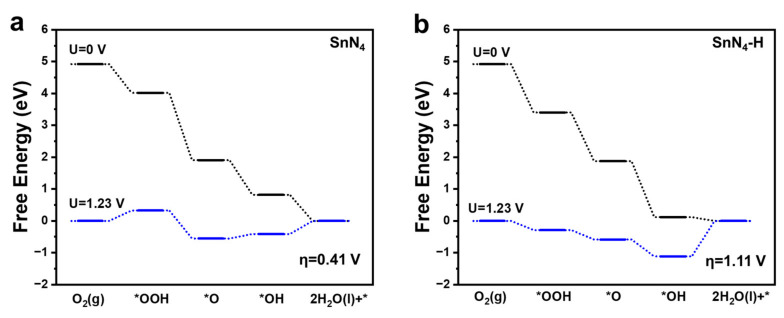
The Gibbs free energy diagrams for the ORR of SnN_4_ (**a**) and SnN_4_-H (**b**) at U = 0 V (black) and U = 1.23 V (blue) under acid conditions.

The computational standard hydrogen electrode (SHE) approach was employed to calculate the abovementioned energy terms for the *O, *OH, and *OOH intermediates. Furthermore, the chemical potential of the H^+^ + e^−^ pair was equal to half the chemical potential of the hydrogen molecule, and the equilibrium potential U_0_ for the four-electron transfer ORR process at pH = 0 was 1.23 V versus reversible hydrogen electrode (RHE) [8]. Furthermore, we identified the specific elementary step that exhibits the highest change in free energy, known as the potential-determining step (PDS), which determines the standard equilibrium potential. This analysis allowed us to pinpoint the rate-limiting step in the electrocatalytic process and gain insights into the underlying mechanisms governing the overall reaction. By understanding the PDS, we can develop strategies to enhance the catalytic performance and improve the efficiency of the system. The PDS represented the unfavorable thermodynamics reaction step on the Sn-N-C surface, and its identification was crucial for understanding and optimizing the ORR process on electrocatalysts. To calculate the overpotential (η), we used the formula η = 1.23 + max(ΔG_(1)_, ΔG_(2)_, ΔG_(3)_, ΔG_(4)_)/e, where e, ΔG_(1)_, ΔG_(2)_, ΔG_(3)_, and ΔG_(4)_ referred to the elementary charge, Gibbs free energy change of the elementary ORR steps (1), (2), (3), and (4), respectively. The illustrated reaction pathways of the associative four-electron were shown in Figures S4 and S5, and the intermediates were all preferentially adsorbed on the Sn atom after structure optimization. The whole process started from the O_2_ adsorption and then hydrogenated to the *OOH, then the first H_2_O was formed by reducing the *OOH, leaving the *O species. The cleaved O* was combined with a proton to form an OH*, then the resulted intermediate was reduced to obtain the product H_2_O. The corresponding free energy diagrams of SnN_4_ and SnN_4_-H at U = 0 V (black) and U = 1.23 V (blue) were revealed in Figure 3. The calculation results revealed that the adsorption of OOH species was the potential-determining step (PDS) of the SnN_4_ catalyst, which was exothermic by 0.82 eV at U = 0 V. For the SnN_4_-H system, the hydrogenation of the *OH species was represented as the PDS which was exothermic by 0.12 eV at U = 0 V. Then, we determined that the η under U = 1.23 V condition of these two catalysts was 0.41 V and 1.11 V, respectively. The lower overpotential observed in SnN_4_ indicated its superior ORR performance compared to catalysts without H-decoration, and it is even comparable to the experimental overpotential of state-of-the-art Pt-based electrocatalysts, which typically exhibit an overpotential of 0.45 V. On the other hand, the H-modified catalytic system showed less effectiveness in the ORR process, possibly due to the strong binding of OH* species with the Sn active site, hindering their release to form H_2_O.

**Figure 4:**
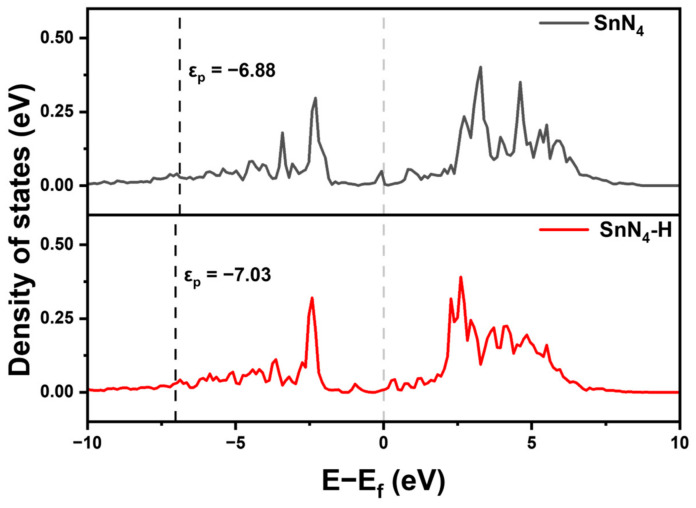
The PDOS of SnN_4_ and SnN_4_-H, the Fermi level was set to 0 eV.

**Figure 5:**
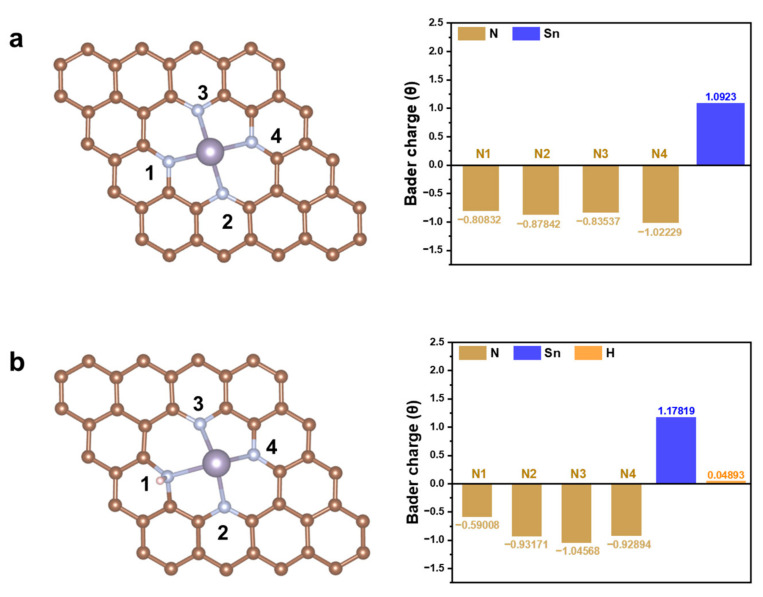
The number of N atoms and Bader charge in SnN_4_ (**a**) and SnN_4_-H (**b**). Color code: brown, C; cyan, N; purple, Sn; white, H.

To gain deeper insights into the superior ORR activity of Sn-SACs, we conducted a detailed analysis of the electronic structure of the SnN_4_ moiety. Specifically, we calculated the partial density of states (PDOS) of the Sn-p and the corresponding p-band center (ε_p_) of SnN_4_ and SnN_4_-H to further unveil the origin of SnN_4_-G catalytic activity. The ε_p_ has been extensively utilized to characterize the interaction behavior between the adsorbate and substrate. The results of this analysis were presented in Figure 4. Notably, the decoration of the H atom influenced the ε_p_ value of the Sn-N_4_ structure, and the ε_p_ of the SnN_4_-H moved away from the Fermi level, which in turn affected its catalytic activity. The lower ε_p_ may result in stronger adsorption of intermediates such as OH*, which well explain the high strength of *OH adsorption of SnN_4_-H and its higher overpotential. Next, the Bader charge analysis of SnN_4_ (Figure 5a) and SnN_4_-H (Figure 5b) indicated that the Sn active center has a positive charge, which was close to that in the bulk SnO (1.19) [7]. The indication was that the coordinated Sn atom was likely in a +2 charge state. The Sn atom in the SnN_4_-H catalyst had more positive charge than the counterpart without H decoration, indicating more electrons transformed from Sn to the N-doped carbon framework. Regardless, the surrounding N2–N4 atoms in SnN_4_-H had more negative charge than their counterparts in the SnN_4_ catalyst. The N atoms decorated with H atoms (N1) exhibited less negative charge in comparison with N atoms without adsorbed H atoms. Upon adsorption of the H atom, the charge distribution in the system can be altered, leading to the transfer of electron density between the H atom and the surrounding atoms. This redistribution of charges can result in the H atom acquiring a positive charge and the Sn atom in SnN_4_-H becoming more positively charged.

## 3. Computational Details

The Vienna Ab Initio Simulation Package (VASP) was employed for all spin-polarized density functional theory (DFT) calculations in this study [21]. The Perdew–Burke–Ernzerhof (PBE) function within the generalized gradient approximation (GGA) was utilized to attain the exchange correlation energy [22]. Ionic cores were modeled using the projector augmented wave (PAW) pseudo-potentials, and a plane wave energy cutoff of 400 eV was selected [23]. Structure optimizations were conducted using an energy convergence criterion of 1 × 10^−4^ eV, with the relaxed ion forces being less than 0.01 eV Å^−1^. The Brillouin zone was sampled using a Monkhorst–Pack k-point mesh of a 3 × 3 × 1 grid for geometric optimization and an 11 × 11 × 1 grid for DOS calculation. A supercell slab of the graphene single layer with dimensions of 4 × 4 was constructed as the substrate model. To prevent interaction between adjacent images, a vacuum space of 15 Å was utilized. To anchor the single Sn atom, we selectively removed two adjacent carbon atoms, and then replaced the four nearest neighboring carbon atoms with N atoms. Subsequently, the single Sn atom was embedded within the structure to create Sn-N_4_ moieties. In addition, we adsorbed an H atom onto the N atoms to form H-modified Sn-N_4_ models. The DFT-D3 method was employed to incorporate van der Waals interactions, with a dispersion correction being implemented [24,25]. The calculational structures were visualized by VESTA [26]. The free energy change (∆G) can be calculated using the computational hydrogen electrode (CHE) model proposed by Nørskov and colleagues [27]:

∆G = ∆E + ∆E_ZPE_ − ∆S + ∆G_pH_ + ∆G_U_

where ΔG, ∆E, ∆E_ZPE_, and ∆S denoted the change in the Gibbs energy for a system, electronic energy, zero-point energy, and entropy change under standard conditions of 298.15 K and 101 kPa, respectively. ∆G_pH_ indicated the change in free energy resulting from a variation in H^+^ concentration. For this work, pH = 1 was taken into account. Meanwhile, ∆G_U_ = −eU was used to describe the change in free energy caused by electrode potential (U). The Gibbs free energies of H_2_ and H_2_O molecules were tabulated in Table S2. Due to the high-spin ground state of the oxygen molecule, its energy calculation presented challenges. Therefore, the free energy of the O_2_ molecule was derived using the following equation:

G_O_2__(g) *=* 2G_H_2_O_(l) − 2G_H_2__(g) *+* 4 *×* 1.23

To evaluate the structural stability of SnNC, the formation energy (E_b_) was calculated as the below equation:

E_b_ = E_total_ − E_Sn_ − E_N-grephene_

where E_total_, E_Sn_, and E_N-grephene_ were the total energies of the catalyst, Sn atom, and defective N-doped graphene, respectively. Finally, the adsorption energy was calculated according to the following equation [28]:

E_ads_ = E_total_ − E_species_ − E_catalyst_

where E_total_ and E_catalyst_ were the total energies of adsorbed species on the SnN_4_ and SnN_4_-H catalyst, respectively, and E_species_ was the energies of the species in the gas phase.

## 4. Conclusions

In conclusion, we carried out a theoretical investigation via DFT calculations of four N-coordinated Sn-SACs (SnN_4_) and H-decorated SnN_4_ catalysts (SnN_4_-H) toward ORR, and we performed the comprehensive studies on the structural and electronic properties. Firstly, the stability of the two catalysts was investigated through energy analysis, revealing that the Sn atoms in both catalysts can be stably anchored to the N-doped graphene without undergoing aggregation or migration. The AIMD simulations further provided valuable insights into the thermodynamic stability of the SnN_4_ and SnN_4_-H catalysts. The electrocatalytic performance of these two catalysts was evaluated by calculating the 4-electron mechanism of the ORR and analyzing the overpotential. The computational analysis provided insights into the efficiency of the catalysts in facilitating the ORR process. The catalyst without H modification exhibited remarkable ORR activity, demonstrated by its lower overpotential of 0.44 V, which was comparable to Pt-based electrocatalysts. Conversely, the H-modified catalyst showed relatively lower performance. To gain a deeper understanding of the factors contributing to the activity of these two SACs, an in-depth analysis of the electronic states was conducted. The theoretical results demonstrated that the original Sn-N_4_ structure holds better ORR performance than its H-modified counterpart. These theoretical findings shed light on the active nature of Sn-N-C electrocatalysts and offer potential avenues for optimizing their performance, enhancing their efficiency, durability, and stability in the operational conditions of PEMFCs. In conclusion, our theoretical investigations have shed light on the active structure of single Sn atom-based ORR electrocatalysts and have identified promising p-block main-group metal SACs that could potentially replace expensive and scarce noble metal catalysts for the ORR. We hope that our findings inspire future research in the design and exploration of other promising SACs for catalytic applications.

## Data Availability

Not applicable.

## Supplementary Materials

The following supporting information can be downloaded at: https://www.mdpi.com/article/10.3390/molecules28145571/s1, Table S1: The binding energy of SnN_4_, SnN_4_-H, Sn-gra; Table S2: The values of Gibbs free energy corrections used for the DFT calculation of non-absorbed gas-phase molecules; Figure S1: The RMSD of SnN_4_ system and Sn-N atoms (a) and SnN4-H system and Sn-N atoms (b); Figure S2: The corresponding O_2_ adsorption structure of SnN_4_; Figure S3: The corresponding O_2_ adsorption structure of SnN_4_-H; Figure S4: The reaction pathway of the associative four-electron SnN_4_; Figure S5: The reaction pathway of the associative four-electron SnN_4_-H.
