# Supplementary material for: Theoretical Insights on ORR Activity of Sn-N-C Single-Atom Catalysts
Source: Molecules. 2023 Jul 21;28(14):5571. doi: 10.3390/molecules28145571 (PMC10384839; doi:10.3390/molecules28145571)
Supplement: Supplementary file 1 [file molecules-28-05571-s001.zip › molecules-2420651-supplementary.pdf]

Theoretical Insights on ORR Activity of Sn-N-C Single-Atom Catalysts

Yuhui Zhang[a], Boyang Li[b] and Yaqiong Su[b,*]

[a] School of Energy and Chemical Engineering, Xiamen University Malaysia, Sepang 43900, Malaysia
[b] School of Chemistry, Engineering Research Center of Energy Storage Materials and Devices of Ministry of Education, National Innovation Platform (Center) for Industry-Education Integration of Energy Storage Technology, Xi'an Jiaotong University, Xi'an, 710049, China
* Correspondence: **authors**: yqsu1989@xjtu.edu.cn (Y. Su)

Table S1. The binding energy of SnN_4, SnN_4-H, Sn-gra.

Entry	Samples	E_b (eV)
1	SnN_4	−5.85
2	SnN_4-H	−4.82
3	Sn-gra	−0.89

Table S2. The values of Gibbs free energy corrections used for the DFT calculation of non-absorbed gas-phase molecules.

Gas	E(DFT)/eV	TS/eV	CvT/eV	ZPE/eV	G/eV
H_2	−6.76	0.05	0.03	0.29	−6.49
H_2O	−14.25	0.13	0.05	0.59	−13.73

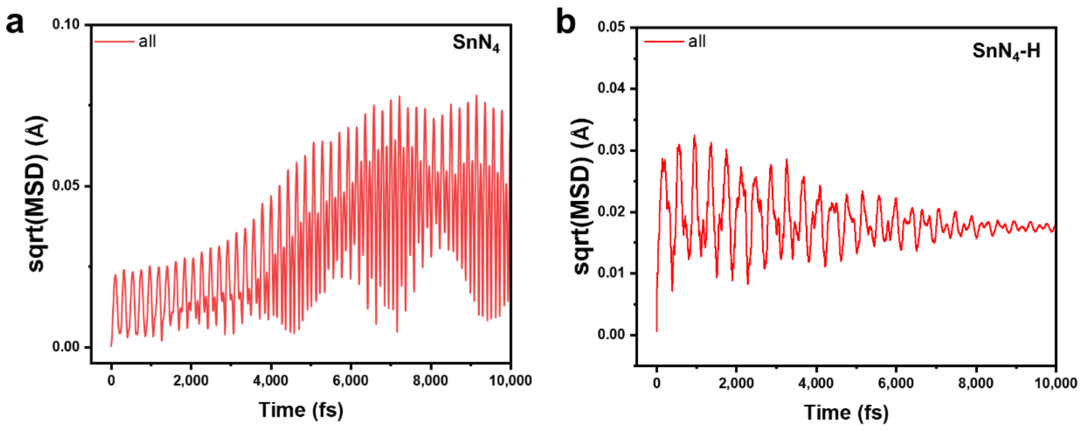

Figure S1. The RMSD of SnN_4 system and Sn-N atoms (a) and SnN_4-H system and Sn-N atoms (b).

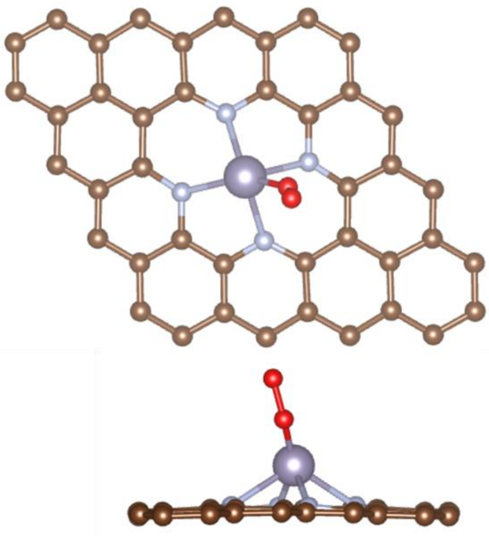

Figure S2. The corresponding O_2 adsorption structure of SnN_4.

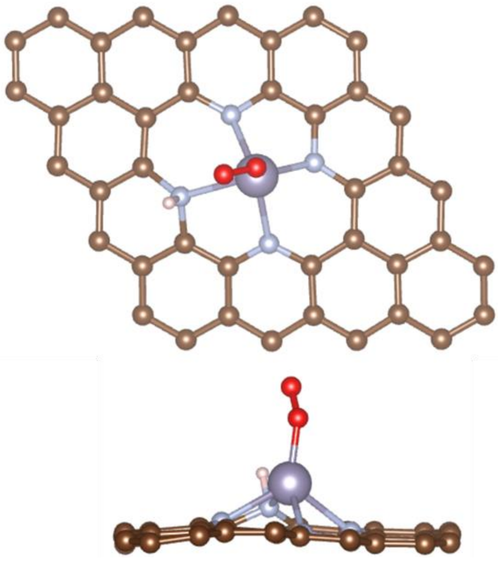

Figure S3. The corresponding O_2 adsorption structure of SnN_4-H.

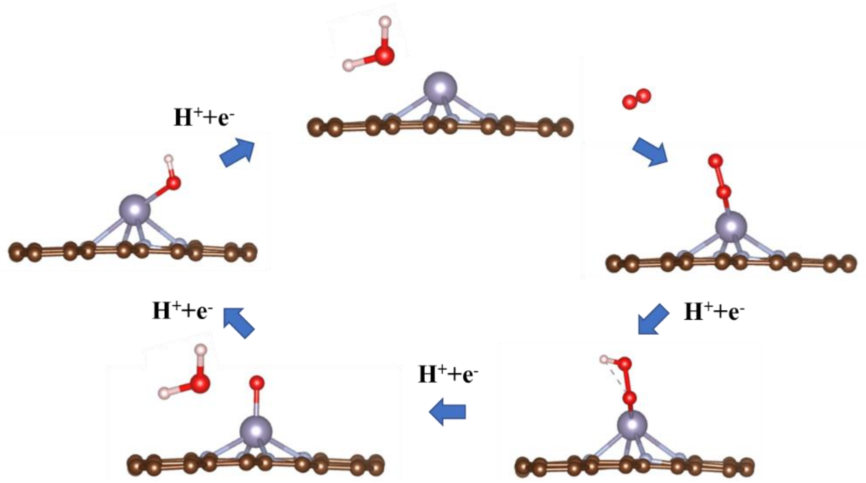

Figure S4. The reaction pathway of the associative four-electron SnN_4.

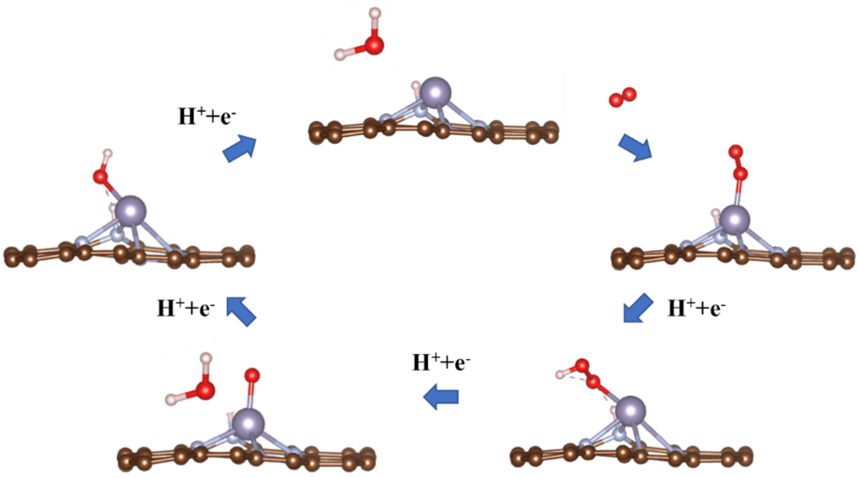

Figure S5. The reaction pathway of the associative four-electron SnN_4-H.
